# *XRCC1* Arg399Gln Polymorphism Confers Risk of Breast Cancer in American Population: A Meta-Analysis of 10846 Cases and 11723 Controls

**DOI:** 10.1371/journal.pone.0086086

**Published:** 2014-01-28

**Authors:** Tao Bu, Li Liu, Yong Sun, Li Zhao, Yang Peng, Shudong Zhou, Lixia Li, Sidong Chen, Yanhui Gao

**Affiliations:** 1 Department of Epidemiology and Biostatistics, School of Public Health and Guangdong Key Lab of Molecular Epidemiology, Guangdong Pharmaceutical University, Guangzhou, China; 2 Department of Prevention and Health Care, The First Affiliated Hospital of Xinjiang Medical University, Urumqi, China; MOE Key Laboratory of Environment and Health, School of Public Health, Tongji Medical College, Huazhong University of Science and Technology, China

## Abstract

**Background:**

In the X-ray repair cross-complementing group 1 (*XRCC1*) gene, a polymorphism, Arg399Gln (rs25487), has been shown to change neoconservative amino acid and thus result in alternation of DNA repair capacity. Numerous studies have investigated the association between Arg399Gln and breast cancer risk in the American population, but yielding inconsistent results. This study aimed to clarify the role of this polymorphism in susceptibility to breast cancer.

**Methods:**

Literatures were searched in multiple databases including PubMed, Springer Link, Ovid, EBSCO and ScienceDirect databases up to April 2013. A comprehensive meta-analysis was conducted to estimate the overall odds ratio (OR), by integrating data from 18 case control studies of 10846 cases and 11723 controls in the American population.

**Results:**

Overall, significant association was observed between the Arg399Gln polymorphism and breast cancer risk under the random-effects model (OR for dominant model = 1.12, 95% CI: 1.02–1.24, *P*_heterogeneity_ = 0.003; OR for additive model = 1.07, 95% CI: 1.01–1.14, *P*_heterogeneity_ = 0.017). Further sensitivity analysis supported the robust stability of this current result by showing similar ORs before and after removal of a single study.

**Conclusions:**

This meta-analysis suggests that the *XRCC1* Arg399Gln polymorphism may significantly contribute to susceptibility of breast cancer in the American population.

## Introduction

Breast cancer is the most common cancer and a predominate cause of cancer related-death in female population worldwide [1]. In 2013, an estiamted 232,340 new cases in women were expected to occurred and 39,620 women were expected to die from breast cancer in the USA [2]. Breast cancer is a complex trait caused by environmental and genetic factors. Multiple environmental factors for breast cancer have been identified, including age at first birth, menarche and menopause, and family history, but the underlying genetic basis remained largely unknown [3].

Base-excision repair (BER), an important DNA repair pathway, is responsible for the repair of base damage resulting from exposure to X-rays, oxygen radicals, and alkylating agents [4], [5], [6]. In the BER pathway, the X-ray repair cross-complementing group 1 (*XRCC1*) gene, encoding a scaffolding protein, involved in the repair of single-strand breaks, the most common lesions in cellular DNA [7]. Molecular studies showed if lacking the *XRCC1* active cell would be hypersensitive to DNA damage. In the *XRCC1* gene, a functional polymorphism, Arg399Gln (rs25487) has been extensively investigated in many cancers [8], [9], [10], [11]. Regarding breast cancer, multiple studies have been conducted to explore the association of this polymorphism and the disease risk in the USA [12], [13], [14], [15], [16], [17], [18], [19], [20], [21], [22], [23], [24], [25], [26]; however, results were inconsistent. For instance, Duell *et al*. suggested that the variant of Arg399Gln might confer increased risk of breast cancer [12], whereas Dawei Bu *et al.* reported no association of this polymorphism and breast cancer [18]. Based on previously published studies, four meta-analysis have been conducted on the Arg399Gln and breast cancer risk [27], [28], [29], [30], but not special in the American population. Maybe due to heterogeneity across different countries, no conclusion has been drawn yet. Unfortunately, in the two most recent meta-analysis [27], [29], some errors in the data extraction have introduced the incorrect results. Herein, we believed that it is essential to conduct an update comprehensive meta-analysis including studies published since 2001 to provide a more precise assessment of the association between the Arg399Gln in *XRCC1* and breast cancer risk in the American population.

## Materials and Methods

### Literature Search

Relevant articles published before April 1st, 2013 were identified through a electronically search in the PubMed, Springer Link, Ovid, EBSCO and ScienceDirect databases using the combination of key words: ‘XRCC1’, ‘polymorphism’, ‘Arg399Gln’, ‘SNP’, ‘variant’, ‘BC’ and ‘breast cancer’. References of retrieved publications were also screened. Disagreements were resolved through discussions between the two authors (Yang Peng and Yong Sun).

### Inclusion and Exclusion Criteria

In our meta-analysis, studies were included if they met the all of the following criteria: (a) case-control studies investigated the relationship between *XRCC1* Arg399Gln and breast cancer risk; (b) patients should be confirmed with histologically breast cancer; (c) studies should provided data about the frequencies of alleles or genotypes. (d) American population is meant that all the inhabitants of America. Meta-analysis, letters, reviews or editorial articles were excluded. If studies shared the same participants, only the one with the largest population or the most complete information was included. If more than one ethnical population were included in one publication, each population was considered separetly. The meta-analysis was conducted according to the guidelines of Preferred Reporting Items for Systemic Reviews and Meta-Analyses statement (PRISMA) [31], as shown in Checklist S1 (http://www.prisma-statement.org).

### Data Extraction

The following data from included studies were extracted independently by two authors (Li Zhao and Yang Peng) into a standardized form: the first author's name, year of publication, ethnicity of participants, study design, sample size, pre- and postmenopausal status, genotyping method, allele and genotype frequencies, study population, sample materials of study participants and evidence of Hardy-Weinberg equilibrium (HWE) in controls. In case of conflicting evaluations, disagreements were resolved through discussions between the authors.

### Quality Assessment of Included Studies

Two authors independently assessed the quality of included studies according to the 9-star Newcastle-Ottawa Scale. The study quality was assessed by the 9-star Newcastle-Ottawa Scale. A full score is 9 stars, and a score ≥6 stars is considered to be high quality. The quality of case-control studies was assessed as follows: adequate definition of cases, representativeness of cases, selection of controls, definition of control, control for the most important factor or the second important factor, exposure assessment, same method of ascertainment for all subjects, and non-response rate. The score of each individual publications was shown in Table S1.

### Statistical analysis

For each study, odds ratios (ORs) and their 95% confidence intervals (CIs) as the metrics of effect size were recalculated for additive, dominant [(Gln/Gln+Arg/Gln) versus Arg/Arg] and recessive [Gln/Gln versus (Arg/Gln + Arg/Arg)] genetic models. For additive model, common homozygotes, heterozygotes, and rare homozygotes were assigned as scores of 0, 1, and 2, respectively, and then ORs per unit score were calculated by comparing between cases and controls in logistic regression model. The χ^2^ based Cochran's *Q* statistic test was employed to test between-study heterogeneity, and heterogeneity was considered significant when *P*<0.1 for *Q* statistic. Heterogeneity was quantified by *I^2^* statistic examining the percentage of heterogeneity (*I^2^* = 0–25%, no heterogeneity; *I^2^* = 25–50%, moderate heterogeneity; *I^2^* = 50–75%, large heterogeneity; *I^2^* = 75–100%, extreme heterogeneity) [32]. For pooling ORs and 95% CIs. A random-effects model using the DerSimonian and Laird's method was applied, with significant evidence of heterogeneity; otherwise, a fixed-effects with Mantel-Haenszel's method was utilized. Furthermore, subgroup analyses were performed by ethnicity, menopausal status, genotyping method and control source, to explore the source of heterogeneity. Sensitivity analysis was also conducted to assess influence of single study on the overall estimate, by sequential removal of individual studies. Publication bias was estimated by funnel plot and Egger's test [33]. All analyses were carried out by using the Stata 12.0 software.

## Results

### Characteristics of included studies

**Figure 1:**
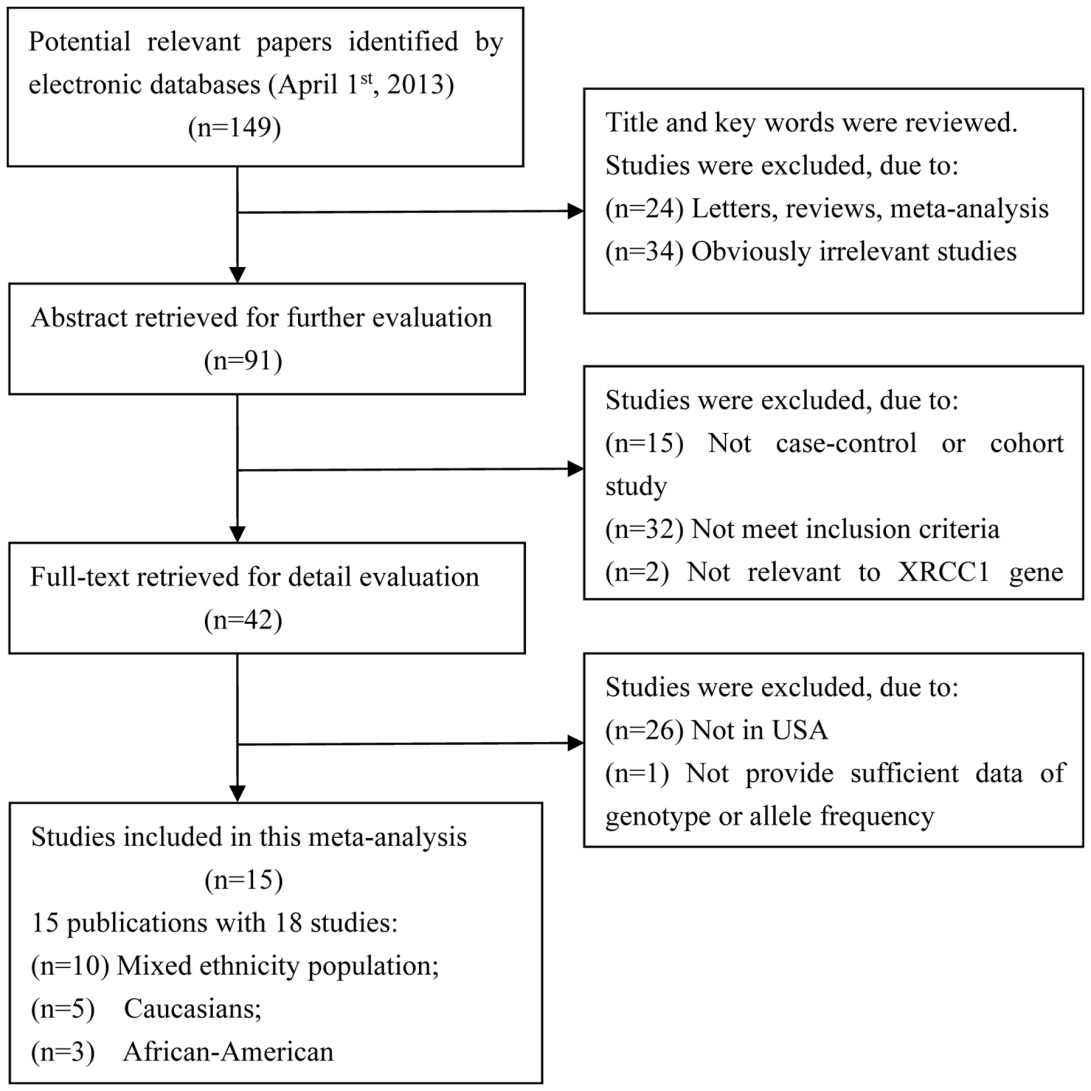
Flow chart of literature search and selection in the meta-analysis.

Figure 1 shows the procedure of study selection. A total of 15 publications with 18 case-control studies of 10846 breast cancer cases and 11723 controls were finally included in this meta-analysis. Among them, 10 studies were conducted in mixed ethnicity population, 3 studies were in the African-American, and 5 studies in Caucasians. The characteristics of individual studies are summarized in Table 1.

**Table 1:** Characteristics of included studies in this meta-analysis.

First author	Year	Ethnicity	Case			Control			Population based	HWE	Sample material	Genotyping methods
			AA	AG	GG	AA	AG	GG				
Duell [12]	2001	African	164	82	7	198	64	4	Population	Y	Blood	PCR-RFLP
Duell [12]	2001	Caucasian	162	175	49	164	58	56	Population	Y	Blood	PCR-RFLP
Smith [13]	2003	Mixed	99	122	30	115	123	29	Hospital	Y	Blood	PCR-RFLP
Smith [15]	2003	Caucasian	70	72	20	119	150	31	Hospital	Y	Peripheral lymphocyte	PCR-RFLP
Han [14]	2003	Mixed	391	460	135	545	616	176	Population	Y	Blood	Pyrosequencing
Shen [17]	2005	Mixed	412	539	116	444	536	130	Population	Y	Blood	PCR-RFLP
Patel [16]	2005	Mixed	196	195	61	194	202	56	Population	Y	Buffy coat	TaqMan Real Time PCR
Bu [18]	2006	Mixed	84	84	22	42	43	10	Hospital	Y	Blood	PCR-RFLP
Zhang [21]	2006	Caucasian	392	1433	1214	360	1173	1054	Population	Y	Mouthwash cytobrush	PCR-RFLP
Brewster [20]	2006	Mixed	108	159	38	126	135	49	Population	Y	Blood	PCR-RFLP
Thyagarajan [19]	2006	Mixed	57	76	60	135	140	47	Population	Y	Blood,normal tissue	PCR-RFLP
Pachkowski [22]	2006	African	536	203	22	493	172	11	Population	Y	Blood	TaqMan Real Time PCR
Pachkowski [22]	2006	Caucasian	504	581	159	480	494	148	Population	Y	Blood	TaqMan Real Time PCR
Ali [23]	2008	Mixed	11	16	13	21	20	7	Population	Y	Normal tissues	PCR-RFLP
Smith [24]	2008	Caucasian	135	141	36	179	181	46	Population	Y	Blood	MassARRAY Sequenome
Smith [24]	2008	African	38	13	1	58	15	1	Population	Y	Blood	MassARRAY Sequenome
Zipprich [25]	2010	Mixed	126	115	30	139	141	43	Population	Y	Blood	SYBR Green PCR
Roberts [26]	2011	Mixed	104	361	417	164	772	814	Hospital	Y	Blood, mouthwash	MassARRAY Sequenome

### Overall meta-analysis

**Figure 2:**
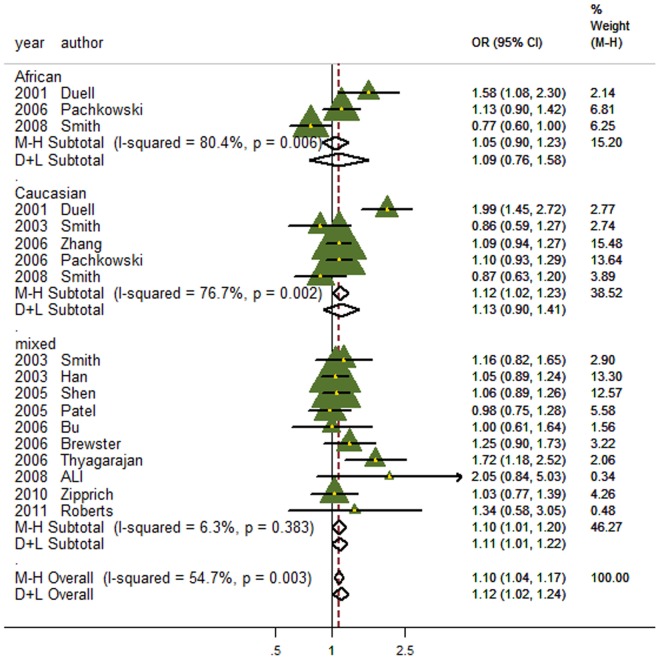
Forest plot of the association between the *XRCC1* Arg399Gln and breast cancer risk for the dominant model.

**Figure 3:**
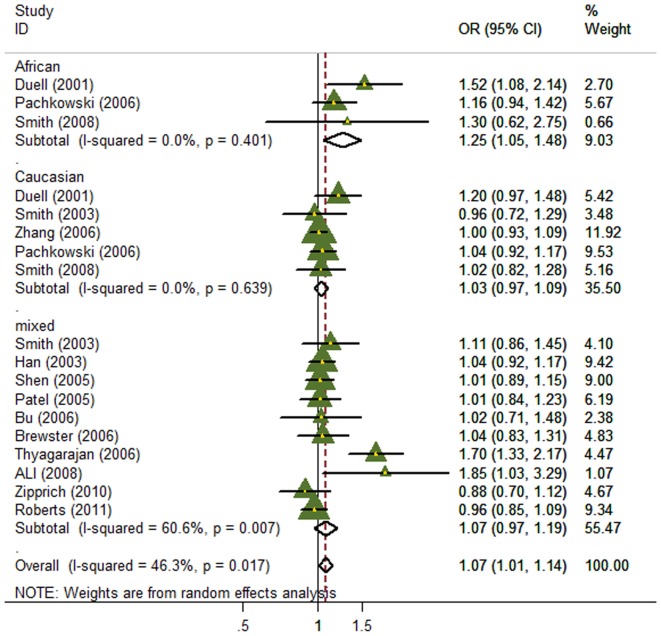
Forest plot of the association between the *XRCC1* Arg399Gln polymorphism and breast cancer risk for the additive model.

In the overall meta-analysis, significant between-study heterogeneity were observed for all genetic models (*P* for heterogeneity = 0.003, 0.003 and 0.017 for dominant, recessive and additive models, respectively), and thus the random-effects model was employed. Significant associations were observed between the *XRCC1* Arg399Gln and breast cancer risk in both of the dominant and additive models (OR for dominant model = 1.12, 95% CI: 1.02–1.24; OR for additive model = 1.07, 95% CI: 1.01–1.14; Figure 2–3), but no association was found in recessive model (OR = 0.95, 95% CI: 0.84–1.08; Figure S1).

### Subgroup meta-analysis

When subgroup analysis was performed by ethnical populations, for the dominant model, only the subgroup with mixed population showed significant association of the Arg399Gln without evidence of heterogeneity (OR = 1.10, 95% CI:1.01–1.20; Figure 2), whereas heterogeneity still existed and no associations were found for both subgroups of African-Americans and Caucasians, possibly due to their relatively small sample size and the moderate effect of this polymorphism under the dominant model. For the additive model, heterogeneity was effectively removed in African-Americans and Caucasians, but only the African-American population showed significant association (OR = 1.25, 95% CI: 1.05–1.48; Figure 3). For recessive model, heterogeneity was effectively removed in African-Americans and Caucasians, but there was still no association in any subgroups.

3 studies provided data according to premenopausal or postmenopausal status (Table S2) [17], [21], [26]. Heterogeneity was effectively removed in postmenopausal subgroup (Figure S2, S3, S4), but no significant association was detected. We considered that based on current limited data, it may lack of sufficient power to detect the real effect of this polymorphism according to premenopausal or postmenopausal status.

When stratified by the genotyping method, the significant was effectively removed in TaqMan Real Time PCR and MassARRAY Sequenome subgroup, but no association was found. In the PCR-RFLP subgroup, heterogeneity was seen (for dominant model: *P* = 0.005, *I^2^* = 62.0%; for additive model: *P* = 0.001 *I^2^* = 62.2%), possibly due to the different sources of controls and ethnicity. Significant association was also seen in this subgroup, with ORs for dominant model and additive model were 1.27 (95% CI = 1.08–1.49) and 1.15 (95% CI = 1.02–1.29), respectively.

Subgroup analysis was also performed by sources of controls (Table 2). The population based subgroup showed significant association, but with evidence of heterogeneity (for dominant model: *P* = 0.001, *I^2^* = 63.4%; for additive model: *P* = 0.006, *I^2^* = 55.5%). No heterogeneity and no significant association were seen in the hospital based subgroup.

**Table 2:** Results of overall analysis and subgroup analysis in this meta-analysis.

Group	Dominant model	Additive model	Recessive model
**Ethnicity**			
Caucasian	1.13 (0.90–1.41)	1.02 (0.96–1.08)	1.08 (0.92–1.27)
African-American	1.09 (0.75–1.58)	**1.24 (1.05–1.47)**	0.56 (0.30–1.03)
Mixed	**1.10 (1.01–1.21)**	1.07 (0.96–1.18)	0.95 (0.84–1.09)
**Menopausal status**			
Premenopausal	1.90 (0.73–4.91)	1.08 (0.98–1.19)	2.24 (0.48–10.35)
Postmenopausal	0.98 (0.86–1.12)	0.93 (0.86–1.01)	1.02 (0.87–1.21)
**Genotyping method**			
PCR-RFLP	**1.27 (1.08–1.49)**	**1.15 (1.02–1.29)**	0.90 (0.70–1.17)
TaqMan Real Time PCR	1.08 (0.96–1.22)	1.04 (1.00–1.13)	0.93 (0.72–1.20)
MassARRAY Sequenome	0.83 (0.68–1.02)	0.98 (0.88–1.09)	0.97 (0.83–1.13)
**Control source**			
Population	**1.14 (1.02–1.28)**	**1.09 (1.01–1.18)**	0.95 (0.80–1.13)
Hospital	1.03 (0.83–1.29)	1.00 (0.89–1.10)	0.95 (0.82–1.10)
**Overall OR**	**1.12 (1.02–1.24)**	**1.07 (1.01–1.14)**	0.95 (0.84–1.09)

### Sensitivity Analysis and Publication Bias

**Figure 4:**
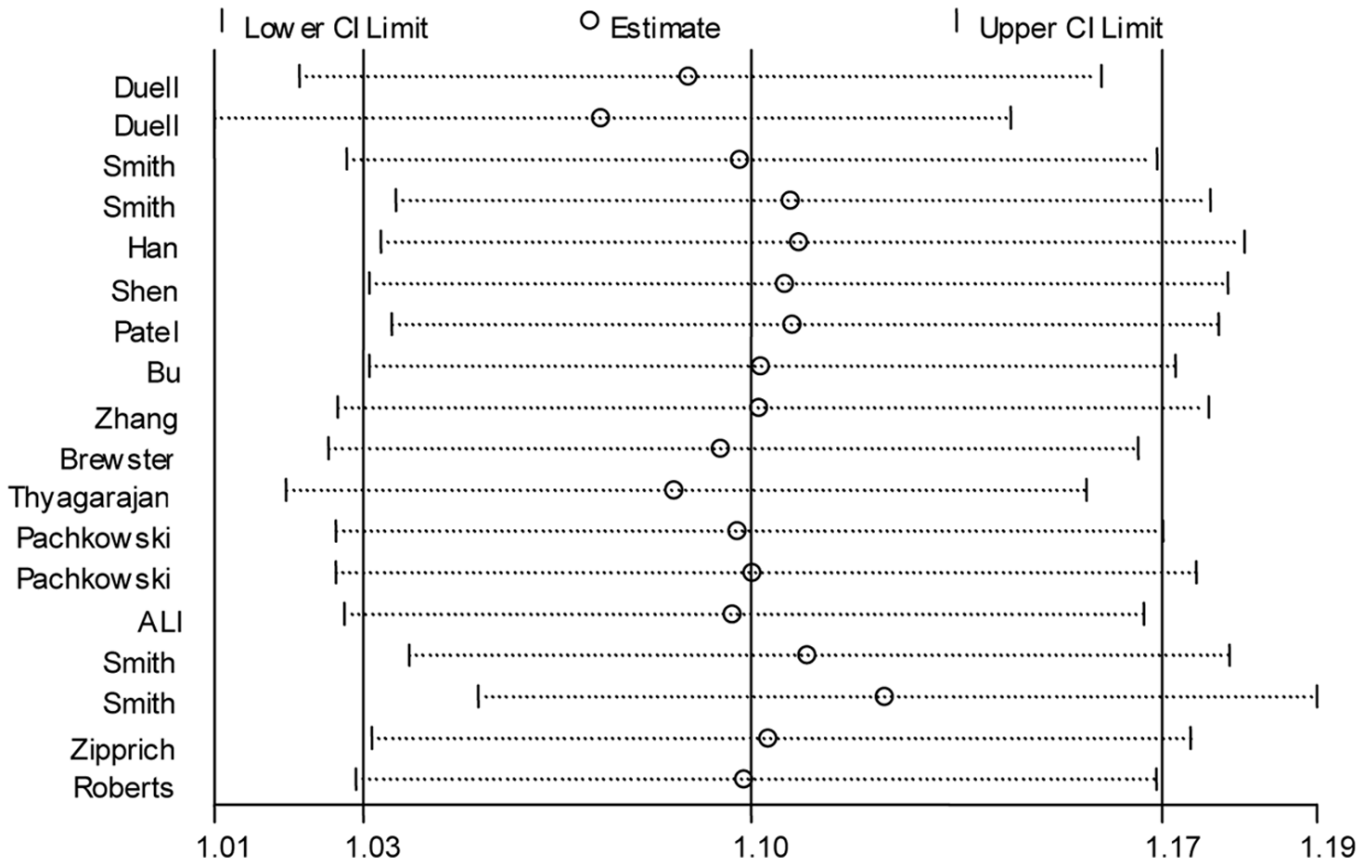
Sensitivity analysis of the association between the *XRCC1* Arg399Gln and breast cancer risk for the dominant model.

**Figure 5:**
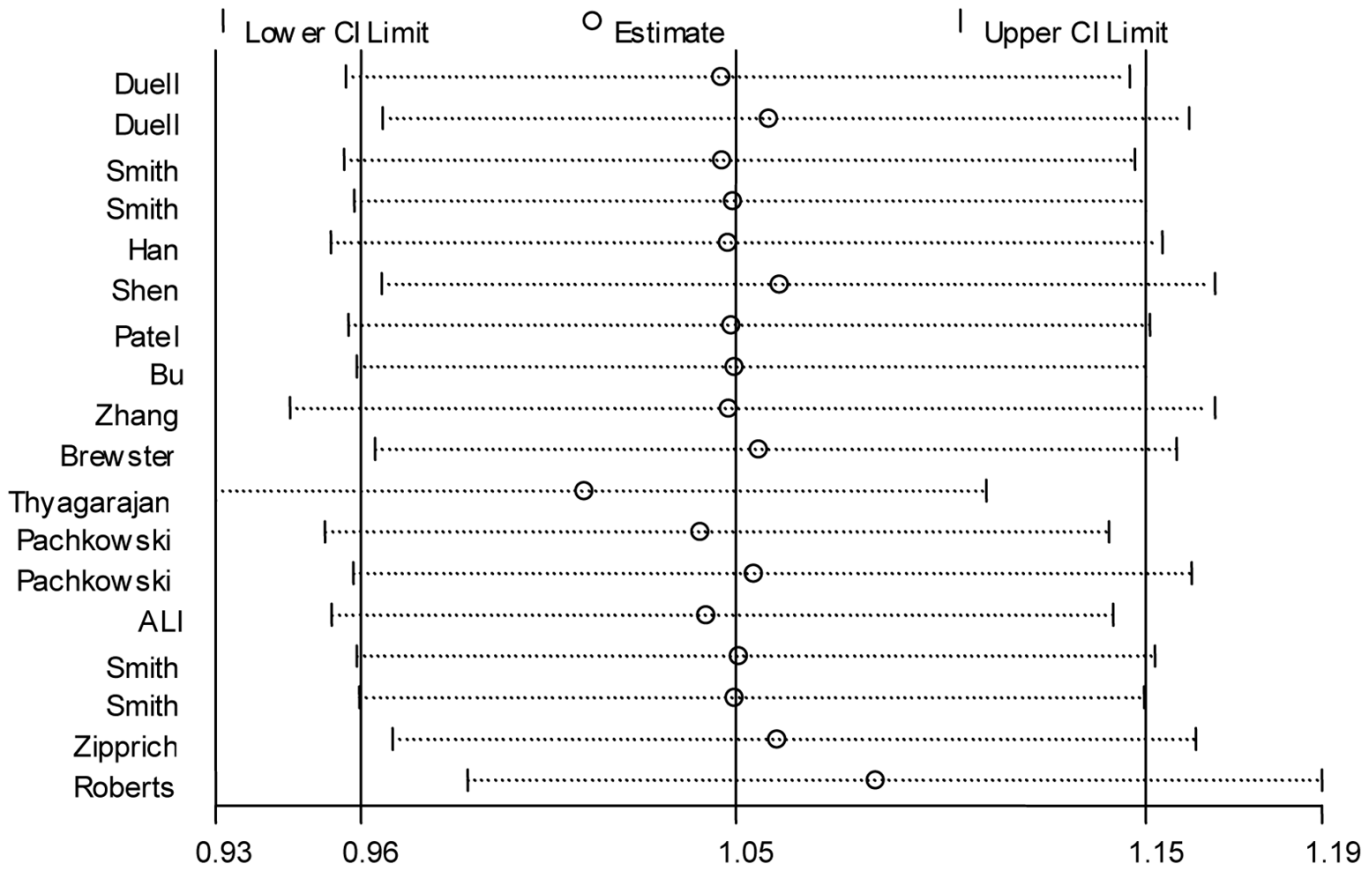
Sensitivity analysis of the association between the *XRCC1* Arg399Gln and breast cancer risk for the additive model.

**Figure 6:**
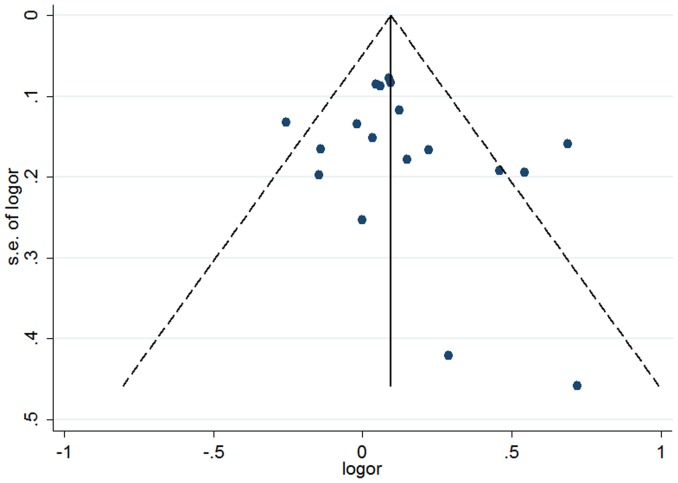
Funnel plot of the association between the *XRCC1* Arg399Gln and breast cancer risk for the dominant model.

**Figure 7:**
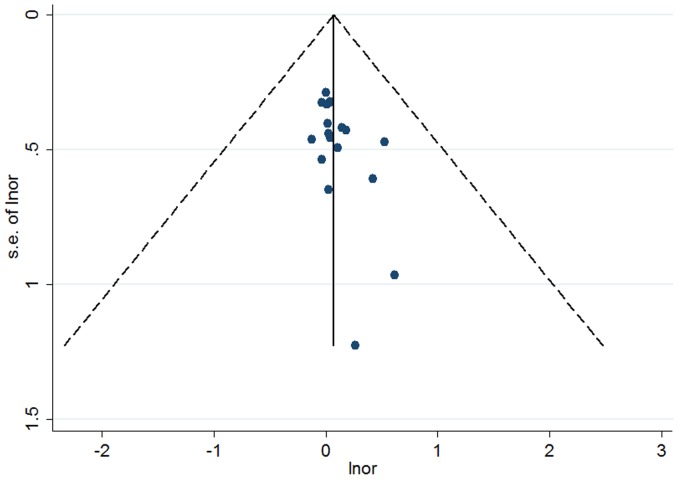
Funnel plot of the association between the *XRCC1* Arg399Gln and breast cancer risk for the additive model.

Given the significant between-study heterogeneity for the Arg399Gln polymorphism, we performed a sensitivity analysis to assess the effects of single study on pooled ORs under a random-effects model (Figure 4–5, Figure S5). The pooled ORs were similar before and after removal of each study, suggesting no single study significantly changes the pooled ORs. As reflected by funnel plots (Figure 6–7 and Figure S6) and Egger's tests, there was no publication bias in the dominant and recessive models (*P* for Egger's test >0.10). For the additive model, a borderline significant publication bias was observed (*P* for Egger's test = 0.04).

## Discussion

This meta-analysis incorporated 18 studies of 10846 breast cancer cases and 11723 controls concerning the Arg399Gln in *XRCC1*. The Arg399Gln variant presented significant association breast cancer risk in the American population. Further sensitivity analysis suggested the stability of the current results, by showing similar ORs before and after sequential removal of single study. This meta-analysis, based on updated published data, has further increased sample size and enlarged the statistical power to reflect the precision effect of the Arg399Gln in breast cancer in the American population.

*XRCC1* plays an important role in the BER pathway, which has been thought of as the predominant DNA-damage repair pathway for the processing of small base lesions derived from oxidation and alkylation damage [34]. The major significance of *XRCC1* in maintaining genomic stability has been raised by high frequency of chromosome deletions or aberrations in the gene mutant cells, and thus the *XRCC1* gene has been posed as a candidate gene for many cancer susceptibility. In the coding region of *XRCC1*, the nonsynonymous polymorphism, Arg399Gln, has caught much attention in breast cancer risk for years. This polymorphism is located in the critical COOH-terminal side of PARP-binding BRCT-domain [35], [36]. The amino acid substitution caused by this variant in the BRCT domian has been shown to completely disrupt the function of XRCC1, and thus may result in reduction of DNA repair capacity [37]. In view of its functional significance, it is biologically possible that the Arg399Gln polymorphism may modulate the risk of breast cancer. As expected, this meta-analysis provides an obvious evidence that the *XRCC1* Arg399Gln polymorphism is significant associated with of breast cancer in the American population. Intriguingly, the significant association was presented in the dominant and additive models, which is inconsistent with the most recent meta-analysis [27], [29]. In these previous meta-analysis, the authors have wrongly extract the control's AA frequency. In the original article by Patel AV *et al.*, the AA genotype frequency in controls was 194; however, in the meta-analysis by Huang Y *et al.* and Wu K *et al.*, it changed to 280, which would influence the accuracy of the pooled analysis. Additionally, Caucasian and African-American assessed in the previous meta-analysis were distinct with our meta-analysis, possibly resulting in the inconsistent result with our meta-analysis.

The association of *XRCC1* with breast cancer has been investigated in many other countries. In China, Liu L *et al.* reported *XRCC1* -77T>C may be a genetic determinant for developing breast cancer [38]. For lung cancer, Liu L *et al.* find that *XRCC1* T-77C could be genetic determinant for prognosis of advanced non-small-cell lung cancer patients treated with platinum-based chemotherapy [39]. Thus, we believed that *XRCC1* Arg399Gln polymorphism maybe also associate with breast cancer.

Nevertheless, significant between-study heterogeneity was seen in this meta-analysis. To explore the source of heterogeneity, we performed subgroup analysis. After stratified by premenopausal or postmenopausal status, heterogeneity was significant removed, indicating that the premenopausal or postmenopausal status may be one source of heterogeneity. According to ethnicity, we found that for recessive and additive models, heterogeneity was effectively removed in Africans and Caucasians, whereas for dominant model, it retained in Africans and Caucasians, but in the mixed population, no evidence of heterogeneity was shown, suggesting ethnical population may also partly explained the heterogeneity of this meta-analysis. With regard to the control source, heterogeneity was removed in hospital-based subgroup, but was detected in population-based subgroup. Furthermore, the results of PCR-RFLP subgroup analysis were similar to population based subgroup analysis. Heterogeneity was also partly explained by population based and genotpying method of this meta-analysis. Moreover, all the included studies showed high quality (≥6 stars) by the 9-star Newcastle-Ottawa Scale, and no publications bias was observed in dominant and recessive models.

Several limitations in this meta-analysis should be figured out. First, in the subgroup analysis by ethnicity and premenopausal/postmenopausal status, the sample size was relatively small and the statistical power might be insufficient. Second, potential sources of heterogeneity in this meta-analysis could include other factors, such as family history of breast cancer, staging of breast cancer, history of begin breast disease. However, due to the limited data, we failed to further explore these factors in the current meta-analysis. Finally, multiple epidemiological studies have demonstrated gene-gene or gene-enviroment interactions may play more important role in cancer development as compared with genetic factors [40], [41]. However, gene-gene interactions and gene-environment interactions could not be appraised in this meta-analysis owing to a lack of special data.

In conclusion, this meta-analysis provided evidence that the *XRCC1* Arg399Gln polymorphism was significantly associated with risk of breast cancer in the American population. Nevertheless, in the future, well-designed studies with large sample sizes will be warranted in diverse populations.

## Supporting Information

Checklist S1 **The PRISMA 2009 Checklist.**
(DOC)

Figure S1 **Forest plot of the association between the** ***XRCC1*** **Arg399Gln and breast cancer risk for the recessive model.**
(TIF)

Figure S2 **Forest plot of the association between the** ***XRCC1*** **Arg399Gln and breast cancer risk of menopausal subgroup for the dominant model.**
(TIF)

Figure S3 **Forest plot of the association between the** ***XRCC1*** **Arg399Gln and breast cancer risk of menopausal subgroup for the recessive model.**
(TIF)

Figure S4 **Forest plot of the association between the** ***XRCC1*** **Arg399Gln and breast cancer risk of menopausal subgroup for the additive model.**
(TIF)

Figure S5 **Sensitivity analysis of the association between the** ***XRCC1*** **Arg399Gln and breast cancer risk the recessive model.**
(TIF)

Figure S6 **Funnel plot of the association between the** ***XRCC1*** **Arg399Gln and breast cancer risk for the recessive model.**
(TIF)

Table S1 **Quality assessment of case–control studies included in this meta-analysis.**
(DOC)

Table S2 **Data of premenopausal or postmenopausal studies.**
(DOC)
