# Supplementary figures and images for: XRCC1 Arg399Gln Polymorphism Confers Risk of Breast Cancer in American Population: A Meta-Analysis of 10846 Cases and 11723 Controls
Source: PLoS One. 2014 Jan 28;9(1):e86086. doi: 10.1371/journal.pone.0086086 (PMC3904848; doi:10.1371/journal.pone.0086086)

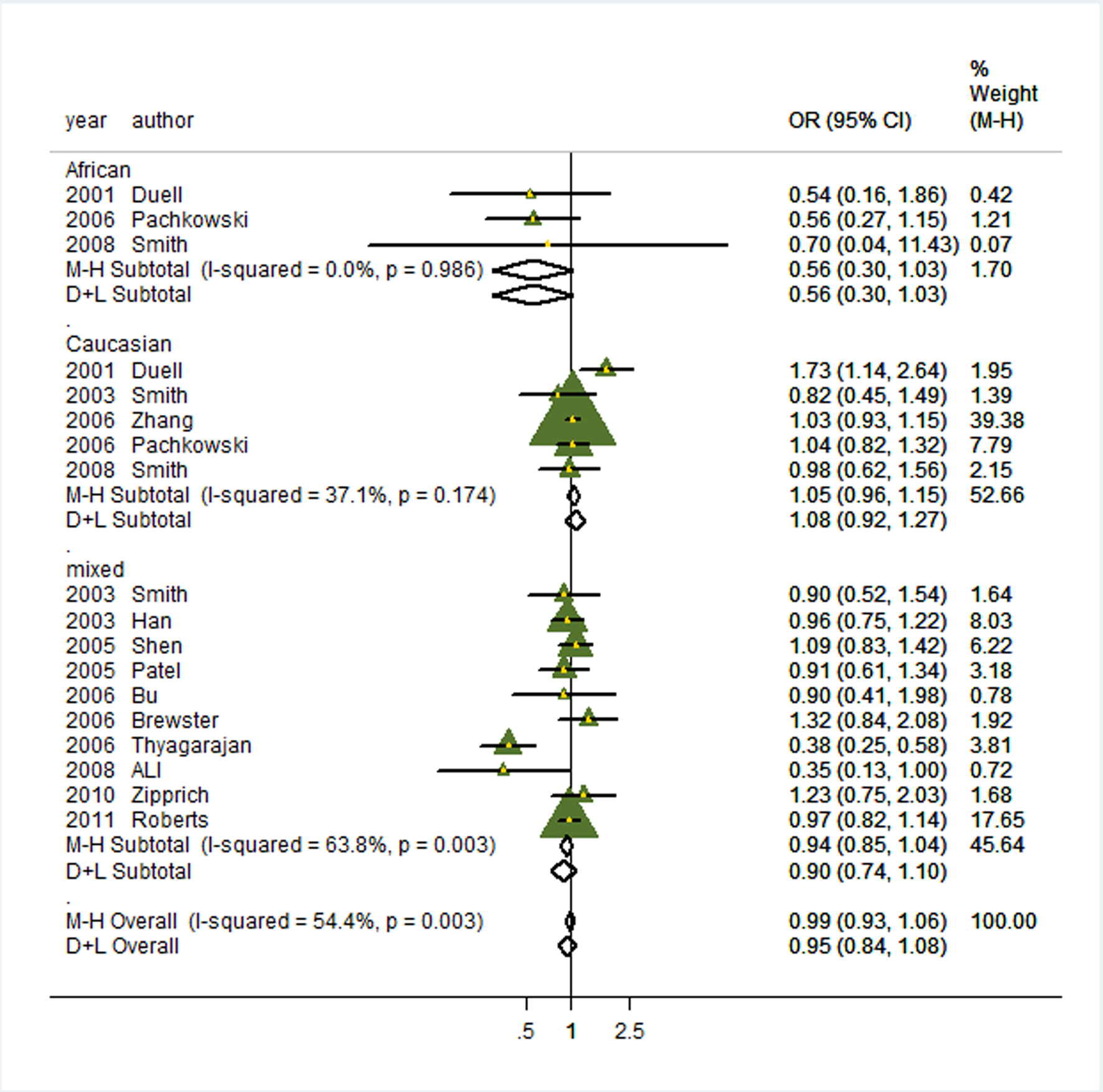

Supplement: Figure S1 — Forest plot of the association between the XRCC1 Arg399Gln and breast cancer risk for the recessive model. (TIF) [file pone.0086086.s002.tif]

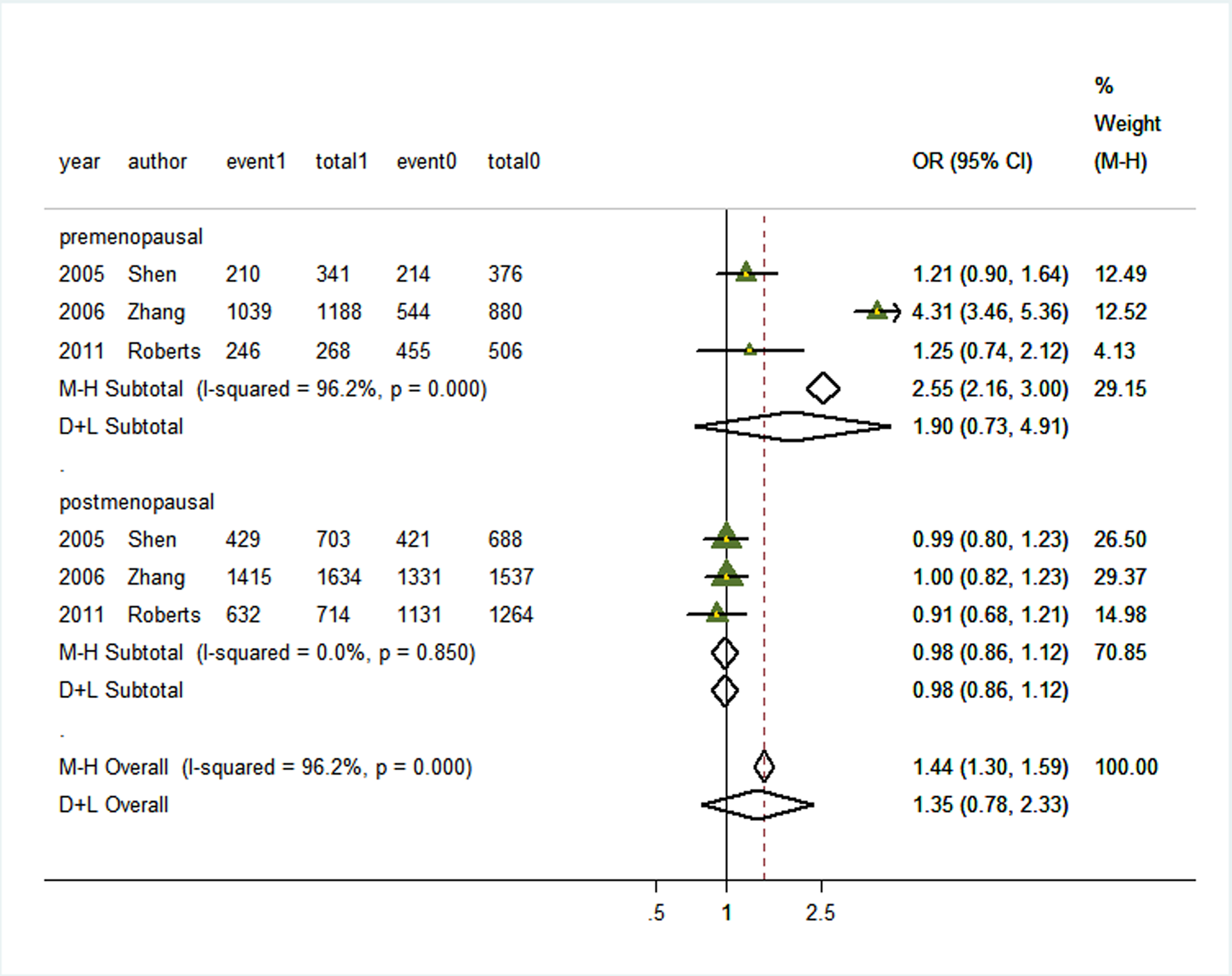

Supplement: Figure S2 — Forest plot of the association between the XRCC1 Arg399Gln and breast cancer risk of menopausal subgroup for the dominant model. (TIF) [file pone.0086086.s003.tif]

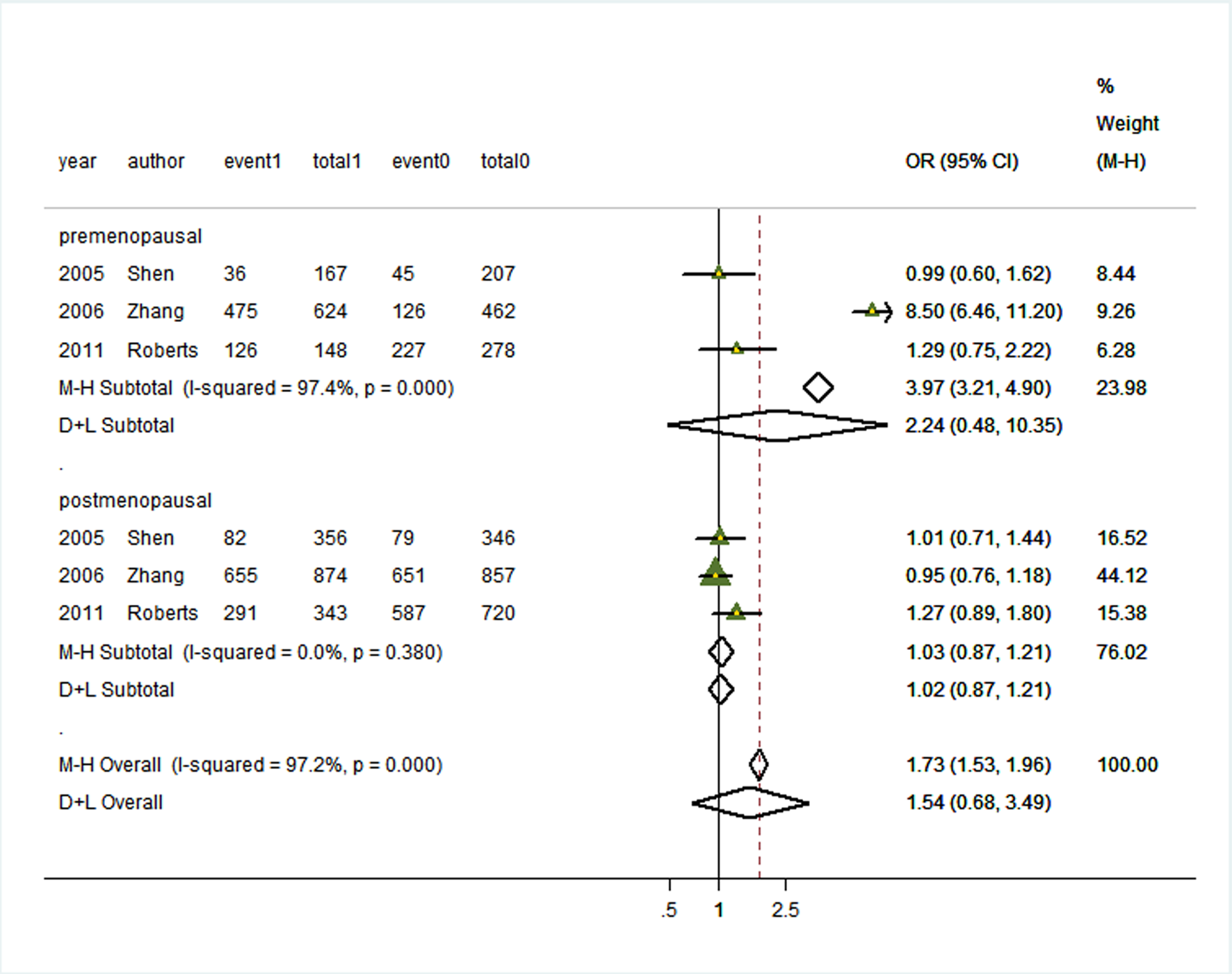

Supplement: Figure S3 — Forest plot of the association between the XRCC1 Arg399Gln and breast cancer risk of menopausal subgroup for the recessive model. (TIF) [file pone.0086086.s004.tif]

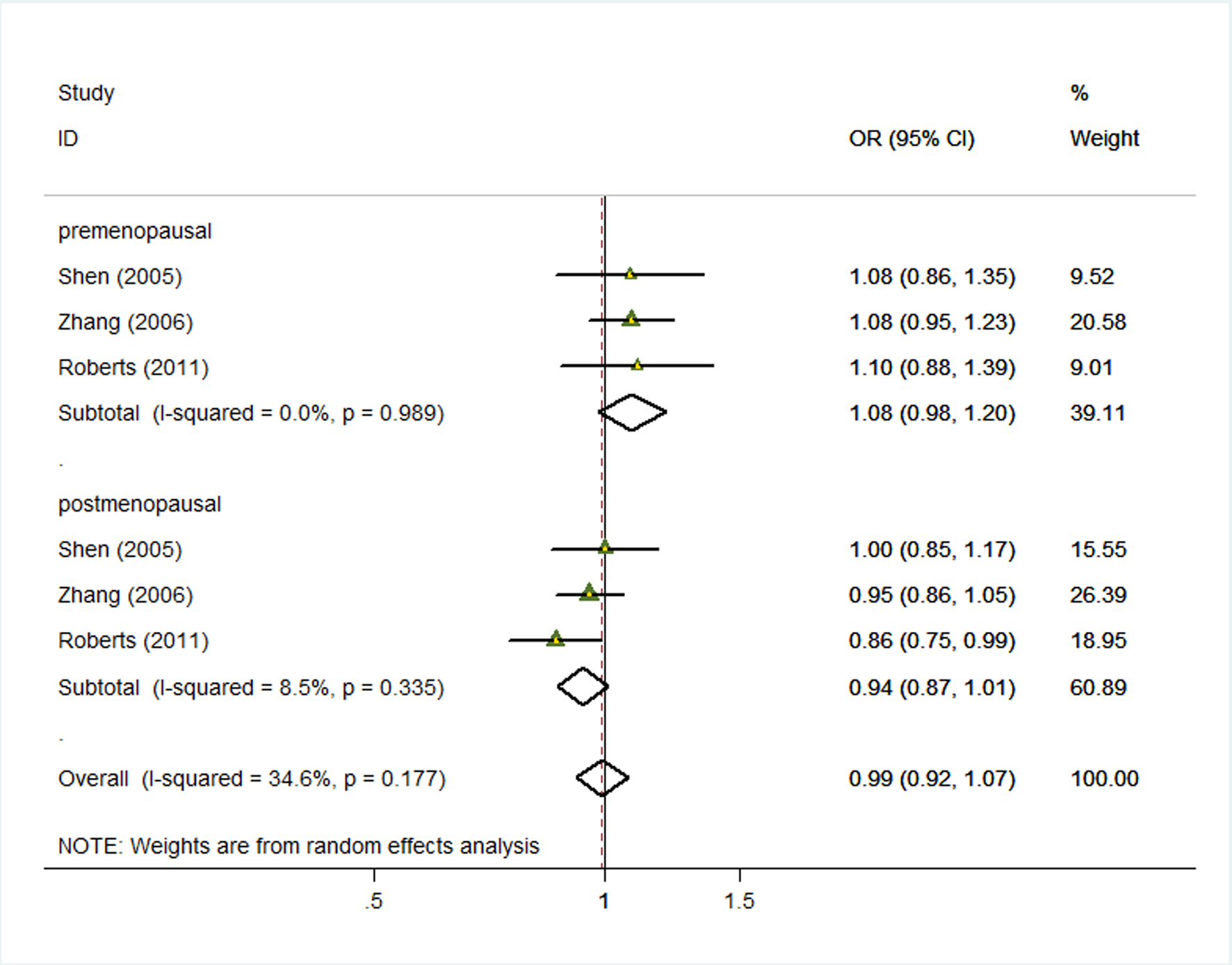

Supplement: Figure S4 — Forest plot of the association between the XRCC1 Arg399Gln and breast cancer risk of menopausal subgroup for the additive model. (TIF) [file pone.0086086.s005.tif]

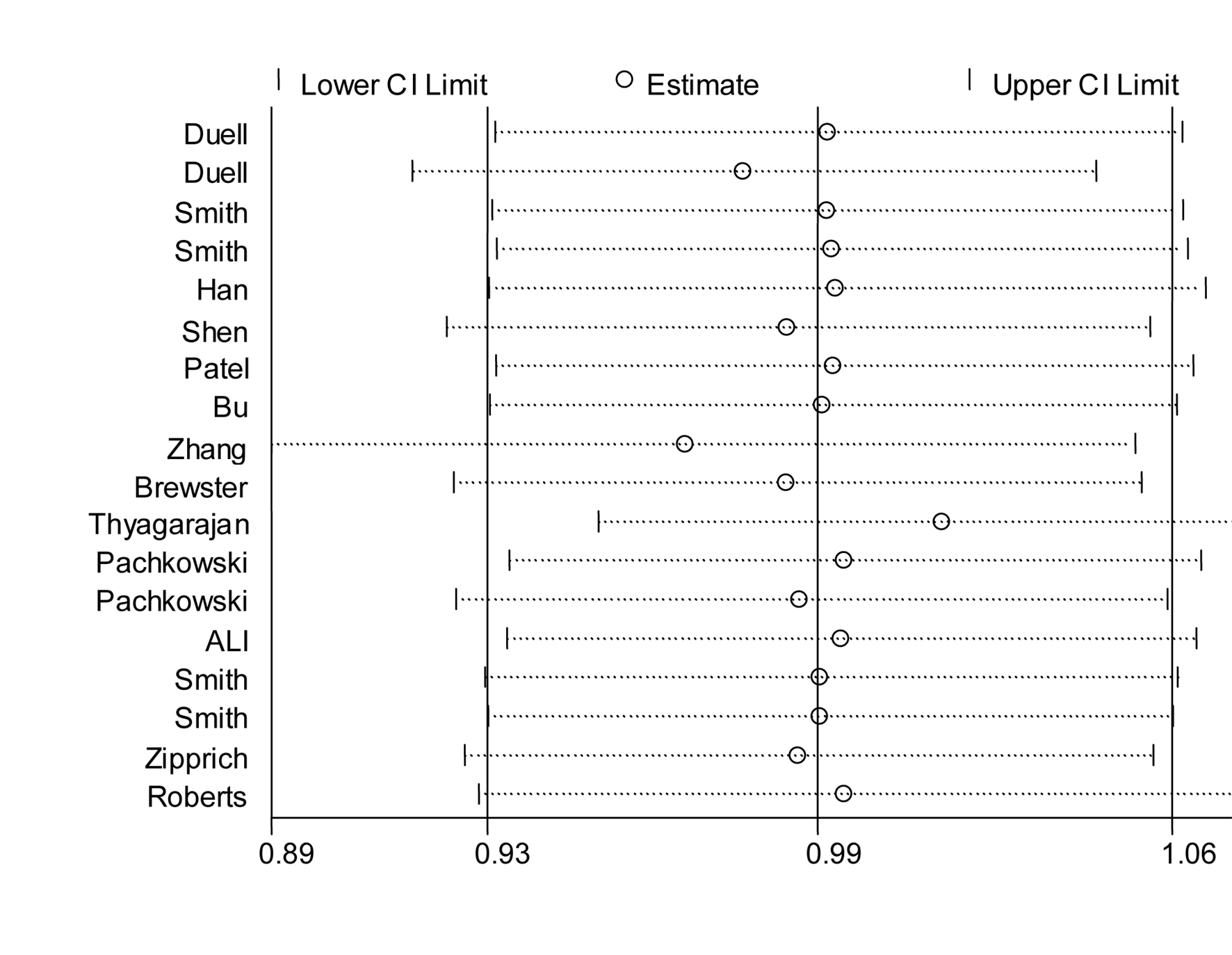

Supplement: Figure S5 — Sensitivity analysis of the association between the XRCC1 Arg399Gln and breast cancer risk the recessive model. (TIF) [file pone.0086086.s006.tif]

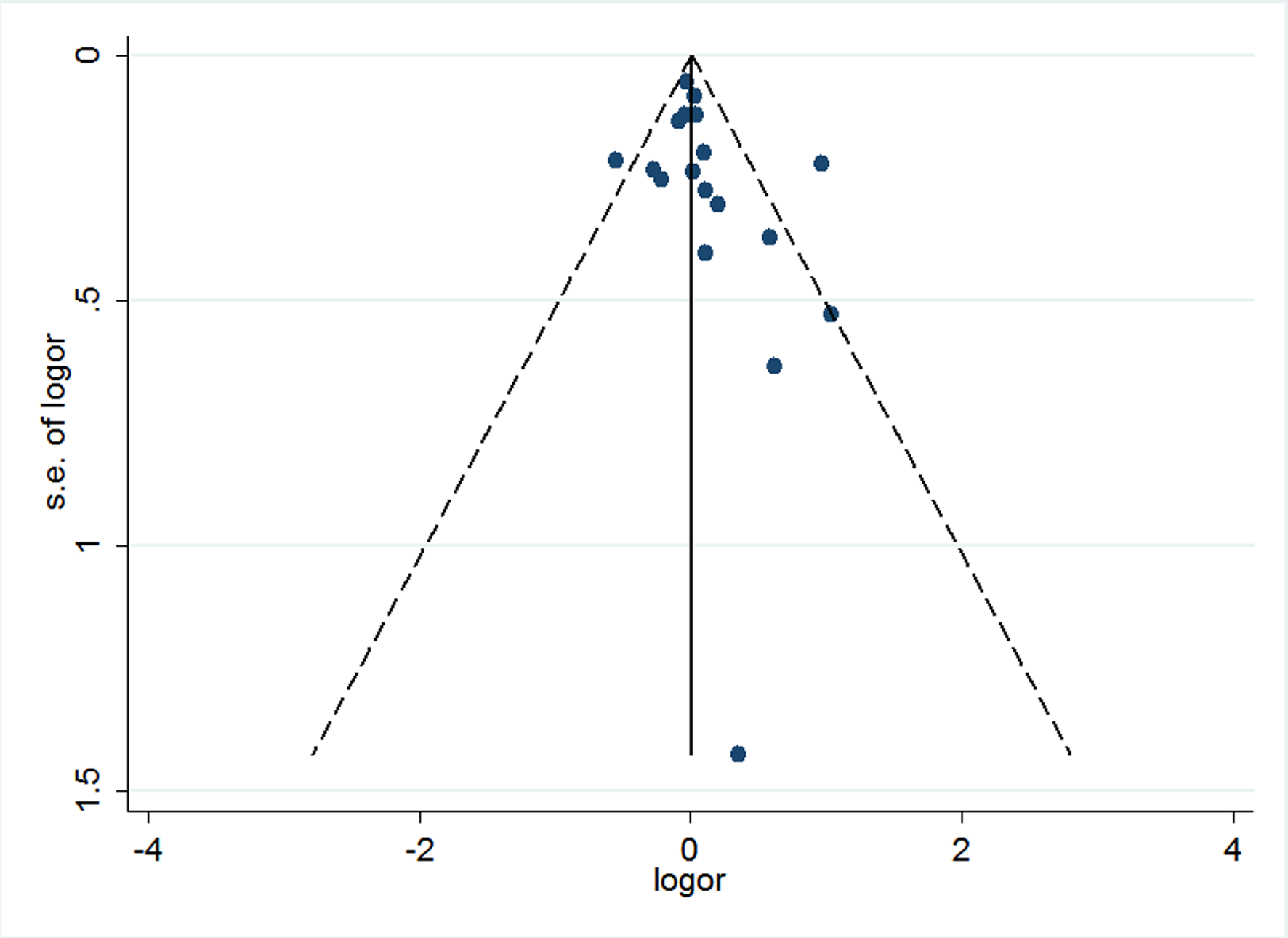

Supplement: Figure S6 — Funnel plot of the association between the XRCC1 Arg399Gln and breast cancer risk for the recessive model. (TIF) [file pone.0086086.s007.tif]
