# Supplementary material for: XRCC1 Arg399Gln Polymorphism Confers Risk of Breast Cancer in American Population: A Meta-Analysis of 10846 Cases and 11723 Controls
Source: PLoS One. 2014 Jan 28;9(1):e86086. doi: 10.1371/journal.pone.0086086 (PMC3904848; doi:10.1371/journal.pone.0086086)
Supplement: Table S1 — Quality assessment of case–control studies included in this meta-analysis. (DOC) [file pone.0086086.s008.doc]

Table S2**.** Quality assessment of case–control studies included in this meta-analysis

| Author | Selection | Comparability | Exposure | Total |
| --- | --- | --- | --- | --- |
| Duell[12] | ★★★★ | ★★ | ★★★ | 9 |
| Smith[13] | ★★★★ | ★★ | ★★ | 8 |
| Smith[15] | ★★★★ | ★★ | ★★ | 8 |
| Han[14] | ★★★★ | ★★ | ★★ | 8 |
| Shen[17] | ★★★★ | ★★ | ★★★ | 9 |
| Patel[16] | ★★★★ | ★★ | ★★ | 8 |
| Bu[18] | ★★★★ | ★ | ★ | 6 |
| Zhang[21] | ★★★★ | ★★ | ★ | 7 |
| Brewster[19] | ★★★★ | ★ | ★★ | 7 |
| Thyagarajan[20] | ★★★★ | ★★ | ★ | 7 |
| Pachkowski[22] | ★★★★ | ★ | ★★ | 8 |
| Ali[23] | ★★★★ | ★★ | ★★ | 8 |
| Smith[24] | ★★★★ | ★ | ★★ | 7 |
| Zipprich[25] | ★★★★ | ★★ | ★★ | 8 |
| Roberts[26] | ★★★★ | ★★ | ★★ | 8 |
