# Supplementary material for: XRCC1 Arg399Gln Polymorphism Confers Risk of Breast Cancer in American Population: A Meta-Analysis of 10846 Cases and 11723 Controls
Source: PLoS One. 2014 Jan 28;9(1):e86086. doi: 10.1371/journal.pone.0086086 (PMC3904848; doi:10.1371/journal.pone.0086086)
Supplement: Table S2 — Data of premenopausal or postmenopausal studies. (DOC) [file pone.0086086.s009.doc]

Table S3. Data of premenopausal or postmenopausal studies.

| First author | Year | Country | Ethnicity | Menopausal | Case | | |  | Control | | |
| --- | --- | --- | --- | --- | --- | --- | --- | --- | --- | --- | --- |
|  |  |  |  |  | AA | AG | GG |  | AA | AG | GG |
| Shen[17] | 2005 | USA | Mixed | Premenopausal | 131 | 174 | 36 |  | 162 | 169 | 45 |
| Zhang[21] | 2006 | USA | Caucasian | Premenopausal | 149 | 564 | 475 |  | 126 | 418 | 336 |
| Roberts[26] | 2011 | USA | Mixed | Premenopausal | 22 | 120 | 126 |  | 51 | 228 | 227 |
| Shen[17] | 2005 | USA | Mixed | Postmenopausal | 274 | 347 | 82 |  | 267 | 342 | 79 |
| Zhang[21] | 2006 | USA | Caucasian | Postmenopausal | 219 | 760 | 655 |  | 206 | 680 | 651 |
| Roberts[26] | 2011 | USA | Mixed | Postmenopausal | 82 | 341 | 291 |  | 133 | 544 | 587 |
